# Locally Advanced Thyroglossal Duct Cyst Carcinoma Presenting as a Neck Mass

**DOI:** 10.1155/2017/7014313

**Published:** 2017-05-09

**Authors:** Niranjan Tachamo, Brian Le, Jeffrey Driben, Vasudev Magaji

**Affiliations:** ^1^Department of Internal Medicine, Reading Health System, West Reading, PA 19611, USA; ^2^Department of Pathology, Reading Health System, West Reading, PA 19611, USA; ^3^ENT Head & Neck Specialists, Wyomissing, PA 19610, USA; ^4^Department of Endocrinology, Reading Health System, West Reading, PA 19611, USA

## Abstract

Thyroglossal duct cyst carcinoma is rare and occurs in just 1% of cases with thyroglossal duct cysts. It is not always possible to distinguish a thyroglossal cyst harboring malignancy from its benign counterparts unless biopsied, thus posing the dilemma. Currently there is no clear consensus on the optimal management of thyroglossal duct cyst carcinoma. Here we present the case of a 69-year-old female who presented with a midline neck mass and dysphagia and was found to have papillary thyroid cancer in the biopsy specimen of the neck mass. She underwent excision of the mass and the thyroglossal duct cyst along with total thyroidectomy; however, the thyroidectomy specimen showed no malignancy. Her lymph node mapping was negative and she is awaiting radioactive iodine treatment.

## 1. Introduction

Thyroglossal duct cyst is a developmental anomaly arising from the failure of thyroglossal duct to involute during embryological development [1]. It is the most common congenital anomaly of the neck [1, 2] and is present in approximately 7% of the general population [1, 3]. Uncommonly, thyroglossal duct cyst carcinoma (TDCC) may be found in 1% of cases, the majority being papillary carcinoma [2, 4]. It may be difficult to ascertain the presence of thyroid carcinoma originating exclusively from a thyroglossal duct cyst as there is no way to distinguish the carcinoma from a benign cyst preoperatively [3]. There is no consensus regarding the optimal management of TDCC.

## 2. Case Presentation

**Figure 1:**
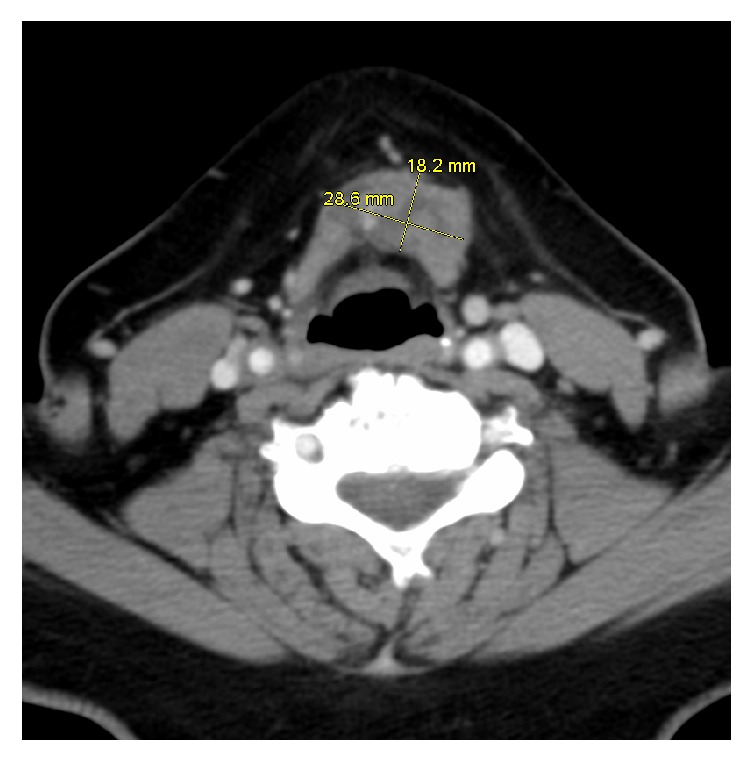
Transverse view of the CT neck that showed presence of a neck mass measuring 28.6 mm × 18.2 mm.

**Figure 2:**
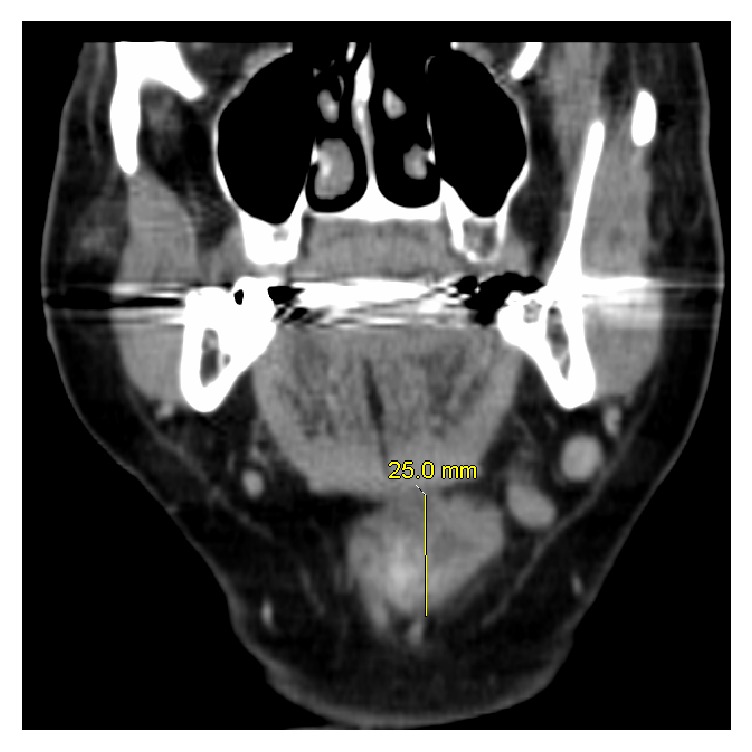
Axial view of the CT neck that showed presence of a neck mass measuring 25 mm in superior to inferior dimension.

A 69-year-old female was seen in the office for a neck lump and dysphagia for 1 year. She denied any fever, night sweats, dysphonia, dysarthria, palpitations, or weight loss. There was no history of radiation to the head and neck. There was no family history of thyroid cancer. The neck mass was 4 × 5 cm, soft to palpation, freely mobile, and located in the midline at the level of the thyroid cartilage. She was evaluated with an ultrasonogram (US) of the neck which revealed a right mid to superior lobe hyperechoic nodule measuring 7 × 3 × 4 mm, with mild increased vascularity, and a left inferior heterogeneous nodule measuring 1.5 × 0.7 × 0.7 cm, along with submandibular lymph nodes, measuring 1.7 × 0.9 × 1.3 cm on the right and measuring 2.1 × 0.9 × 1.1 cm on the left. Computed tomography (CT) of the neck revealed a lobulated soft tissue mass at the level of the left side of the hyoid bone measuring 1.8 × 2.9 cm with no evidence of erosion of the hyoid bone or thyroid cartilage (Figures 1 and 2). Fine needle aspiration biopsy of the neck mass revealed papillary thyroid cancer. The patient underwent excision of the mass and thyroglossal duct cyst via a Sistrunk procedure with total thyroidectomy.

**Figure 3:**
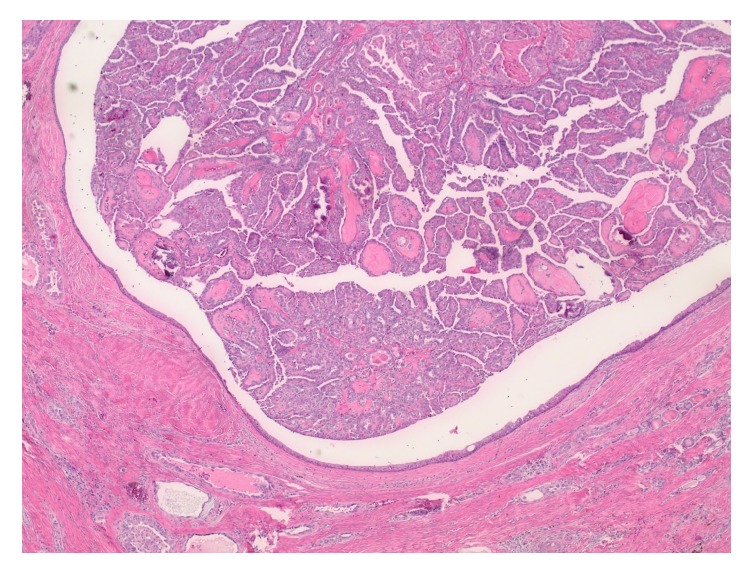
Central neck soft tissue demonstrating a cystic structure, within which is a proliferation of fibrovascular cores (H&E stain, 40x original magnification).

**Figure 4:**
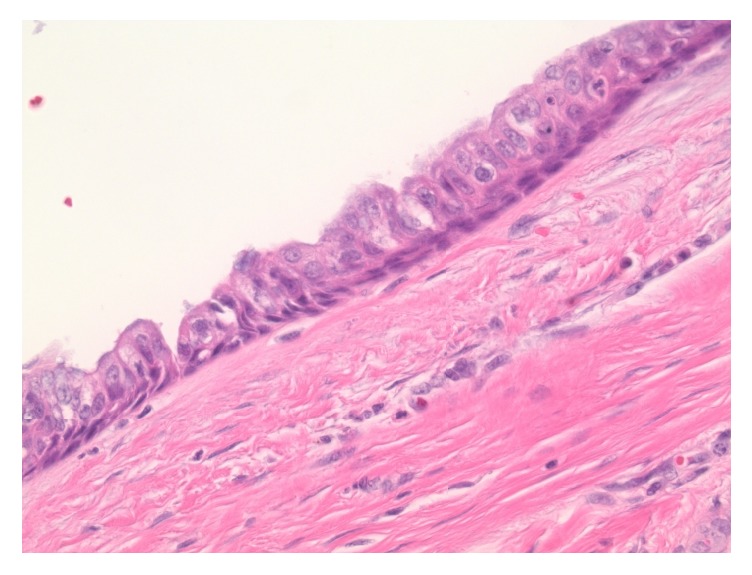
The cystic structure is lined by respiratory-type epithelium, consistent with thyroglossal duct cyst (H&E stain, 400x original magnification).

**Figure 5:**
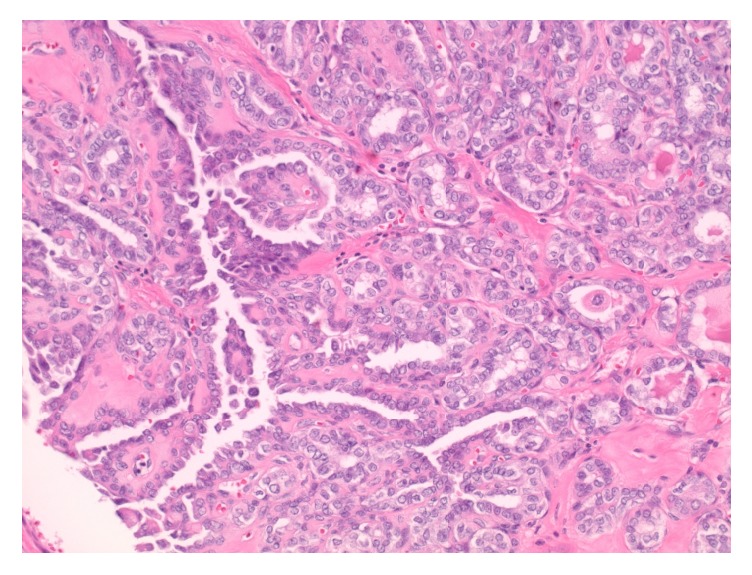
The proliferative tumor is characterized by complex and arborizing papillary fronds (H&E stain, 200x original magnification).

**Figure 6:**
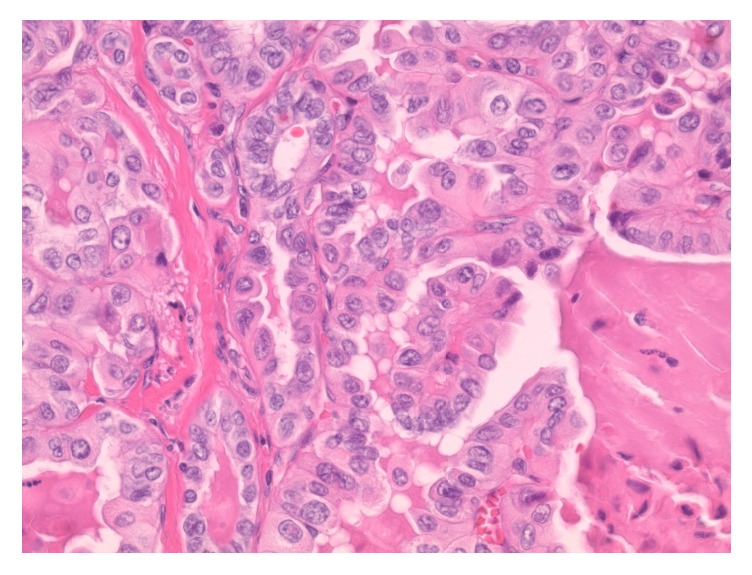
High-power view of the tumor, demonstrating cells with nuclear grooves, peripheralization of chromatin, and some prominence of nucleoli, diagnostic of papillary thyroid carcinoma (H&E stain, 400x original magnification).

On histologic examination, within the central neck soft tissue there is a cystic structure, within which is a proliferation of papillary fronds (Figure 3). The cyst is lined by respiratory-type epithelium, consistent with thyroglossal duct cyst (Figure 4), while the intraluminal proliferation, which extends into adjacent soft tissue, demonstrates complex and arborizing fibrovascular cores lined by epithelioid cells (Figure 5). These cells are characterized by nuclear grooves with pseudoinclusions, peripheralization of nuclear chromatin, and some prominence of nucleoli (Figure 6). The morphologic features are diagnostic of papillary thyroid carcinoma. As there is no demonstrable tumor in the total thyroidectomy specimen concurrently reviewed, the tumor is deemed to have arisen from the thyroglossal duct cyst. Her lymph node mapping was negative and she is planned for treatment with radioactive iodine.

## 3. Discussion

The thyroid gland develops from the endodermal thickening at the base of the tongue and descends via the thyroglossal tract into its final position in the anterior neck inferior to the thyroid cartilage [2, 5, 6]. This thyroglossal tract usually disappears by 10th week of gestation [2, 5]. However, it fails to involute in 7% of cases [1, 4]. Cystic degeneration of this persistent duct forms a thyroglossal duct cyst, which is usually benign [5, 6]. So far, thyroglossal duct cyst is the most common congenital cause of neck swelling and accounts for more than 75% of midline neck swellings in childhood [6]. Between 1.5 and 45% of these cases show the presence of ectopic thyroid tissue [6].

Thyroglossal duct cyst carcinoma (TDCC) is a rare phenomenon and occurs in just 1% of cases of thyroglossal duct cysts [2, 4]. TDCC is more common in females than in males. The mean age of presentation is 6 years in the pediatric population and 38 years in the adult population [4]. Well differentiated thyroid carcinomas account for 95% of TDCC, with papillary cancer being the most common. However, anaplastic thyroid carcinoma and squamous cell carcinoma arising from the cyst have been reported [1].

The origin of TDCC is not entirely clear, whether it arises de novo from the native thyroid tissue in the cyst wall or as a metastasis from the thyroid gland [1, 5]. Interestingly, medullary carcinoma in thyroglossal cyst has never been reported in literature, suggesting a de novo origin of TDCC [5]. In our case, the presence of papillary carcinoma in the thyroglossal duct cyst despite normal thyroid tissue on surgical pathology supports the origin of the cancer from the thyroglossal duct remnant rather than metastasis. The most common papillary carcinomas have indolent growth and an excellent prognosis [1]. In one study, the 5- and 10-year survival rate were found to be 100% and 95.6%, respectively, with no disease-related deaths reported [7].

Most of the time, it is impossible to distinguish the thyroglossal duct cyst harboring malignancy from the benign counterparts [4–6]. Malignancy should be suspected if the thyroglossal duct cyst is hard, irregular, fixed, rapidly growing, and associated with palpable neck lymph nodes [2, 4]. Ultrasonogram (US) and fine needle aspiration cytology (FNAC) of the cyst may help diagnose TDCC preoperatively [5]. Calcifications within the cyst and/or regional calcifications suggest papillary carcinoma while a solid component suggests malignancy. However, TDCC may exist even with a normal US and FNAC [5].

The optimal management of TDCC is still controversial. In the study by de Tristan et al., TDCC was found to be present in just 1.4% (4 out of 352) of thyroglossal duct cysts, and all of them were papillary carcinoma. Three of the 4 patients underwent total thyroidectomy (TT) but none were found to have a second carcinoma in the thyroid suggesting that thyroidectomy was unnecessarily performed [8]. In this regard, some authors suggest tailoring the surgical strategy as per the risk group stratification [5].

Accordingly, it is recommended that the Sistrunk procedure (SP) alone be performed in low risk situations with a clinical and radiologically normal thyroid gland [4, 5]. The low risk situation is defined as age < 45 years, size < 4 cm, no prior radiation exposure, no soft tissue invasion, no distant or lymphatic metastasis, and no aggressive tumor histology [4, 5]. The addition of total thyroidectomy and radioactive iodine ablation (RAI) is done in high risk patients and in cases with positive surgical margins [5]. The rationale is that there was no significant overall survival benefit of TT and RAI in low risk patients. However, Bakkar et al. in their studies found a high rate (62.3%) of concomitant thyroid cancer and recommend routine addition of TT to SP [5]. Moreover, coexisting thyroid cancers may go undetected in US thyroid and the size of a TDCC may not be a reliable predictor of coexisting thyroid carcinoma. In this context, routine TT and RAI would eliminate the latent or residual disease and would positively impact the disease-free survival [5]. This treatment strategy would further facilitate the detection of persistent or recurrent disease based on serum thyroglobulin measurement and RAI scan [5]. However, prophylactic neck dissection is not recommended for papillary TDCC as occult node positivity is common in papillary carcinomas of the thyroid gland, and it does not prognosticate disease recurrence or disease-specific survival [5]. In our case, the patient was an elderly female with muscle invasion at the time of diagnosis, necessitating the need for extensive surgery.

## 4. Conclusion

TDCC is uncommon and is usually diagnosed postoperatively. The majority of the cases are papillary carcinoma and have a good prognosis with long-term survival. However, controversy still exists regarding the optimal management of TDCC. Whether total thyroidectomy with radioactive ablation and lymph node dissection should be performed even in low risk cases is still not clear, necessitating the need for more prospective studies.
